# Surge of human astrovirus type 1 infection in summer 2022 in Korea

**DOI:** 10.1017/S0950268824000980

**Published:** 2025-01-06

**Authors:** Su-Kyung Lee, You La Jeon, Eun-Jung Cho, Han-Sung Kim, Jae-Seok Kim, Wonkeun Song, Hyun Soo Kim

**Affiliations:** 1 Department of Laboratory Medicine, Hallym University College of Medicine, Chuncheon, Korea; 2 Laboratory Medicine, Green Cross Laboratories, Youngin, Korea

**Keywords:** Astrovirus, genotype, Korea, PCR

## Abstract

As astroviral infection rapidly increased in the summer of 2022 in Korea, this study aimed to determine the cause and genotype of astroviruses during this period. From January to December 2022, we tested 43,312 stool samples from patients with acute gastroenteritis utilizing multiplex PCR to detect HAstV. For the HAstV-positive samples, we determined the genotypes of the HAstVs by PCR and sequencing. The monthly positive rate from 2015 to 2022 showed a notable and abrupt increase of HAstV infection between June and August 2022, peaking at 9.8% in July 2022. The annual positivity rate of HAstV remained at 2–3% between 2015 and 2019, and then decreased to 0.5% in 2020, followed by an increase to 1.5% in 2021 and 3.6% in 2022.The genotyped astroviruses in 2022 were all identified as HAstV-1 type, and the nucleotide identity% among them was >99%. The GenBank accession number for the strain genetically closest to the strains identified in our study was ON571597.1, which was HAstV-1 isolated from Pingtan in 2019. Our results provide recent epidemiological data on HAstVs in Korea. The decline and surge in astrovirus positivity in recent years may be related to the COVID-19 pandemic.

## Introduction

Human astroviruses (HAstVs) remarkably contribute to acute gastroenteritis (AGE) in vulnerable populations [1]. The overall pooled prevalence is 3.48%, with a prevalence of 3.85% in children <5 years old, 3.51% in those aged 5–18 years, and 2.37% in individuals >18 years [2]. Among children under 5, regional prevalence varies widely (0–19.4%) [3, 4].

HAstVs (family *Astroviridae*; genus *Mamastrovirus*; species *Mamastrovirus 1–19*) are unenveloped, positive-sense, single-stranded RNA viruses with a genome length of approximately 6.2–7.8 kb. This genome comprises three open reading frames (ORFs): ORF1a, ORF1b, and ORF2 [5]. ORF1a and ORF1b produce nonstructural proteins, including an RNA-dependent RNA polymerase, while ORF2 encodes the precursor of capsid [3]. The genotypic classification of HAstV includes the classic HAstV (HAstV-1–8), novel HAstV Melbourne (HAstV-MLB) (MLB1–3), and novel HAstV Virginia (HAstV-VA) (VA1–5) types [6].

Classic HAstV type 1 (HAstV-1) is the most prevalent genotype worldwide; HAstV-2–8, HAstV-MLB, and HAstV-VA have lower prevalence [1, 2, 7–9]. Their distributions and prevalence vary based on region, period, and age group [2]. In Korea, several studies have explored the positivity rates and genotypes of HAstVs and reported astroviral infection prevalence of 1–4% [10, 11]. After astrovirus infections surged during the summer of 2022, we sought to identify the cause of this surge and prevalent specific genotypes.

## Methods

### Samples

Between January and December 2022, 41,587 and 1,725 stool samples from the Green Cross Laboratory (GC Labs) and Hallym University Dongtan Sacred Heart Hospital, respectively, were tested for astrovirus using multiplex PCR (Allplex GI-Virus Assay, Seegene, Seoul, Korea). These samples were collected by hospitals from patients suspected of having AGE. GC Labs is a specialized clinical testing institution that conducts laboratory tests upon requests from medical institutions of various sizes nationwide. Hallym University Dongtan Sacred Heart Hospital is a 650-bed sized hospital located in the metropolitan area near Seoul. The median age of the 43,312 patients was 9.0 years, with a range of 0 to 101 years.

Forty-three astrovirus-positive stool samples (1–9 samples/month) were collected and preserved at temperatures below −70 °C until use. Since the multiplex PCR reagent for astrovirus detection used in this study can only detect the classic HAstV types 1–8, we analysed 100 astrovirus-negative samples from patients <5 years of age in July and August 2022 to detect novel HAstV-MLB and HAstV-VA types. To examine changes in monthly astrovirus positivity rates by year, 202,769 astrovirus test results were collected from two laboratories from 2015 to 2022. From these results, the number of astrovirus-positive samples and positivity rates were investigated. The Institutional Review Boards (IRB) of Hallym University Dongtan Sacred Heart Hospital (IRB No. 2023-06-009) and Green Cross Laboratory (GCL-2023-1043-01) approved this retrospective study, with waivers of informed consent.

### Genotyping

Genotyping involved reverse-transcription PCR and subsequent sequencing [11]. Stored stool samples positive for HAstV PCR underwent thawing, followed by viral RNA re-extraction. The generated cDNA was derived from RNA using the Invitrogen SuperScript III First-Strand Synthesis System (Thermo Fisher Scientific Inc., Waltham, MA, USA) and used in subsequent PCR reactions with DNA AmpliTaq Gold Taq (Applied Biosystems GmbH, Weiterstadt, Germany). We used a gene-specific primer set, PreCAP1 (5′-GGACTGCAAAGCAGCTTCGTG-3′) and 82b (5′-GTGAGCCACCAGCCATCCCT-3′), which targets the *ORF2* gene and generates a 719-bp PCR product [12]. For 100 astrovirus-negative stool samples, a pan-astrovirus primer set – SF0073 (5’-GAYTGGACWCGATTTGATGGTAC-3′) and SF0076 (5′-CTGGCTTAACCCACATTCC-3′), which targets the *ORF1b* gene and generates a 409-bp product – was used to detect HAstV-MLB (MLB1–3), HAstV-VA (VA1–5), and classic HAstV [13]. A positive control (RNA extracted from the sample of a HAstV-positive patient and a synthetic plasmid control) and a negative control (RNase-free water) were co-amplified to assess false-negative and false-positive results, respectively. The PCR products were visualized via agarose gel electrophoresis, purified using the QIAquick PCR Purification Kit (Qiagen Inc., Valencia, CA, USA) according to the manufacturer instructions, and sequenced using ABI BigDye Terminator v3.1 Cycle Sequencing Kits (Applied Biosystems Inc., Foster City, CA, USA) on an ABI PRISM 3703XL Analyzer (Applied Biosystems). HAstV genotypes of the obtained sequences were determined using the Basic Local Alignment Search Tool (BLAST).

### Phylogenetic analysis

Phylogenetic analysis was performed to assess the genetic relationships among the HAstVs detected in this study, their closest strains, and reference strains of each genotype in GenBank. The accession numbers of these closest strains were ON571597.1, MG932591.1 (the closest strain of GC-060), and OQ968307.1 (the closest strain of DT 22–32). Reference strains of HAstV1 (NC 001943), HAstV2–V8 (MK 059950–MK 059956), VA1–VA5 (NC 013060, GQ 502193, NC 019026, NC 01929, KJ 656124), MLB1–MLB3 (NC011400, NC 016155, NC 019028), MK472054 (D-1312 December 2016), and MK430063 (D-1415 July 2017) detected in Korea during 2016–2017 were also included to construct phylogenetic trees [11]. The HAstV sequences were aligned using MEGA version 11 [14]; phylogenetic trees were then constructed using the maximum-likelihood method and Tamura–Nei substitution models. The construction involved 1,000 bootstrap replications using MEGA version 11.

### GenBank accession numbers

The GenBank accession numbers for the nucleotide sequences of the strains detected in this study are PP779595–PP779607 and PP782785–PP782511.

## Results

### Positive rates

**Figure 1.**
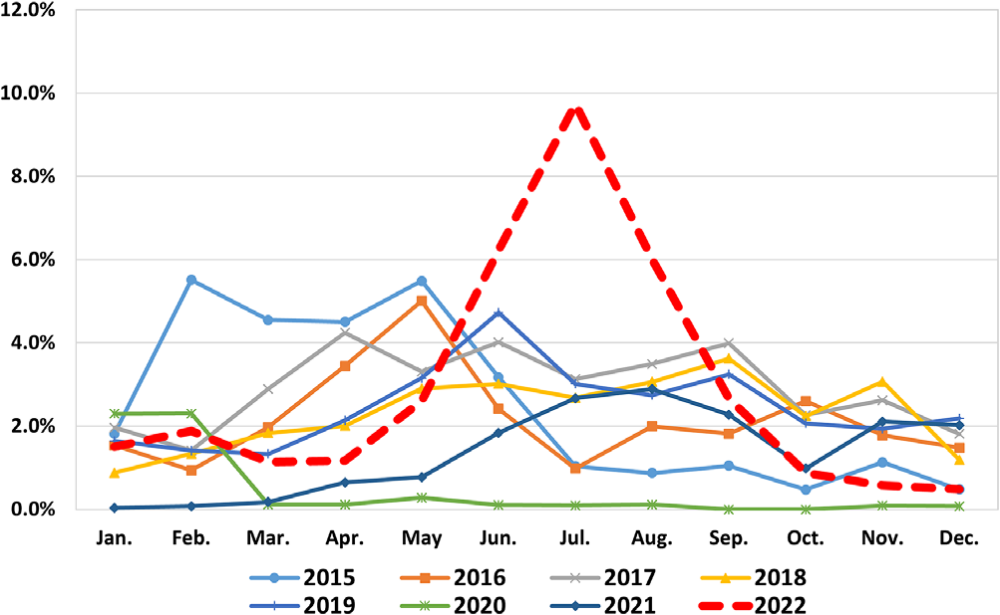
Monthly HAstV positivity rates from January 2015 to December 2022 (n = 202,769). The figure represents the monthly HAstV positivity for the two laboratories from 2015 to 2022. Up until 2021, there was no evident seasonality, and the peak positivity rates remained below 6%. A notable and abrupt increase in HAstV positivity rates was observed between June and August 2022. Abbreviation: HAstV, human astrovirus.

Table 1 illustrates HAstV positivity rates by patient age. Among samples of patients with AGE in this study, 6.1% of those aged 1 year or younger and 8.2% of those between the ages of 2 and 5 years were diagnosed with HAstV infection. In age groups over 6 years, the positive rate for HAstV was lower compared to those under 5 years (1.2% vs. 7.3%). In 2022, the average positivity rate was 3.6%; the age of HAstV-positive patients was 0–90 (median: 3; average age: 6) years. Monthly positivity rates from 2015 to 2022 are shown in Figure 1. The annual positivity rate of HAstV remained steady, at 2–3%, between 2015 and 2019. However, it considerably dropped to 0.5% in 2020, followed by an increase to 1.5% in 2021 and 3.6% in 2022. Up until 2021, there was no evident seasonality, and the peak positivity rates remained below 6%. A notable and abrupt increase in HAstV positivity rates was observed between June and August 2022: June 2022 (6.2%, 324/5214), July 2022 (9.7%, 547/5617), and August 2022 (6.0%, 283/4712). Neither the novel HAstV-MLB nor HAstV-VA was detected in the 100 stool samples that were negative for astrovirus PCR, collected in July and August 2022.

**Table 1.** Age distribution of HAstV positivity in patients from two laboratories in 2022

Age (years)	HAstV positivity rate at GC Labs% (positive no./tested no.)	HAstV positivity rate at HUDSHH% (positive no./tested no.)
≤1	6.1 (467/7,676)	8.6 (6/70)
2–5	8.2 (737/8,980)	17.1 (7/41)
6–10	3.7 (147/3,985)	7.5 (3/40)
11–20	0.8 (26/3,113)	0 (0/76)
21–30	1.4 (25/1,797)	1.4 (2/140)
31–40	2.9 (34/1,171)	1.6 (2/125)
41–50	0.6 (7/1,191)	0.7 (1/144)
51–60	0.5 (8/1,653)	0 (0/183)
≥61	0.3 (25/8,551)	0.2 (2/906)
Unknown	2.1 (74/3,470)	
Total	3.7 (1,550/41,587)	1.3 (23/1,725)

Abbreviations: HAstV, human astrovirus; HUDSHH, Hallym University Dongtan Sacred Heart Hospital.

### Genotype and phylogenetic analysis

**Figure 2.**
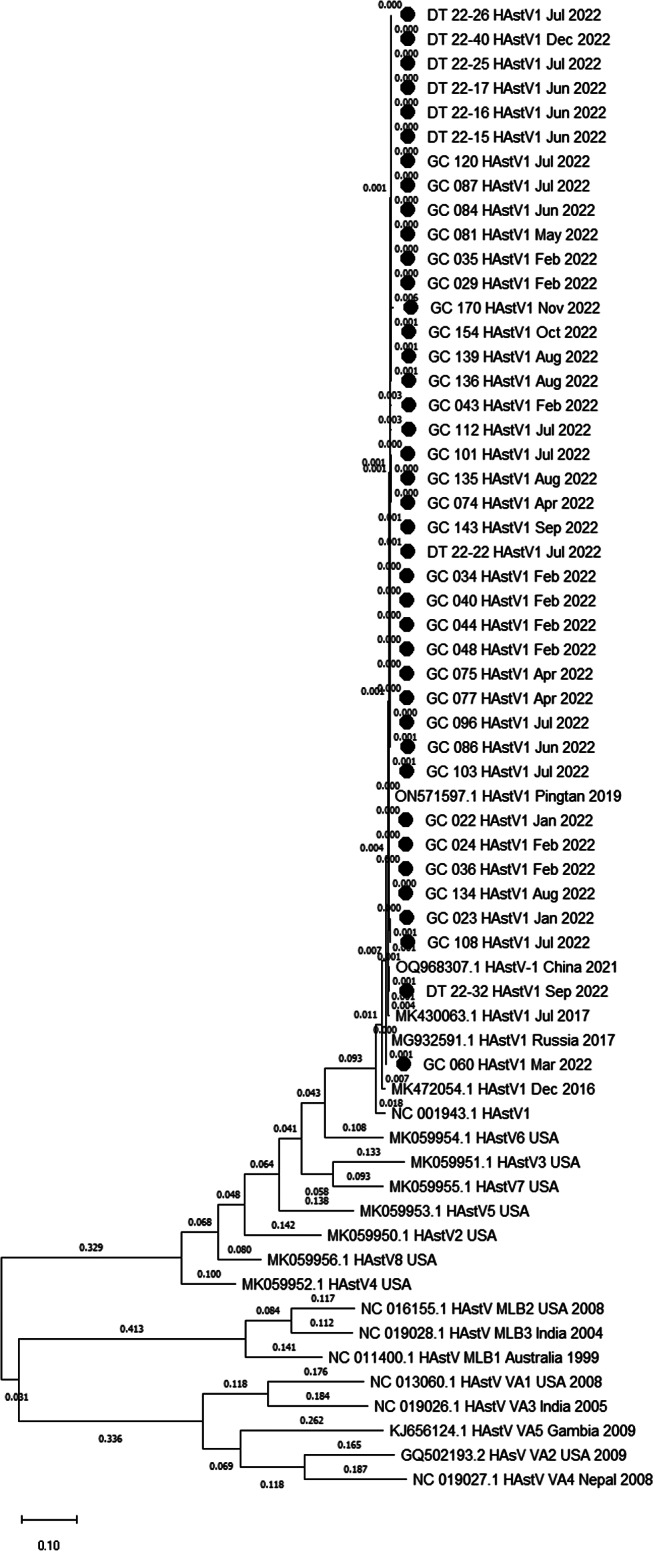
Phylogenetic analysis of HAstVs collected in 2022 and reference strains in GenBank. Phylogenetic trees were constructed using the maximum-likelihood method, using MEGA version 11. The tree is drawn to scale, with branch lengths measured in the number of substitutions per site. Branch lengths are shown above the branches. Black circles indicate the strains from this study. All HAstVs detected in 2022 belong to the same HastV-1 type, exhibiting minimal sequence variation. Abbreviation: HAstV, human astrovirus.

Of the 43 HAstV-positive samples collected, 41 were successfully genotyped, while 2 samples did not yield PCR products via the genotyping primer sets used. Genotyping results indicated that all HAstVs detected in 2022 belonged to the same HAstV-1 type, exhibiting minimal sequence variation (Figure 2). In 39 of the 41 HAstV-positive cases in 2022, the strain exhibiting the closest nucleotide sequence resemblance through a BLAST search was ON571597.1, which was HAstV-1 type isolated from Pingtan in 2019. In our samples, these strains had >99% identity (ranging from 99.1% to 100%, with ≤6 different nucleotide sequences) with the nucleotide sequence of ON571597.1, and three samples had an identical sequence to ON571597. The two remaining samples showed the closest similarity to the strain MG932591.1 (GC-060, identity 99.4%) and OQ968307.1 (DT 22–32, identity 99.6%). Other types of reference strains showed significant genetic distances between the strains detected in this study (Figure 2).

## Discussion

Owing to increased astrovirus positivity rates between June and August 2022 compared to previous periods, we investigated potential shifts in the prevalent epidemic strains. Genotyping results revealed that all instances were HAstV type 1, with minimal sequence variation.

The prevalence rates of HAstV among patients with AGE vary by region, testing methods, and timing of testing, ranging from 0% to 61% [3, 4, 15, 16]. According to a meta-analysis of studies that used PCR to detect HAstV, the estimated global overall pooled prevalence is approximately 3.48% [2]. Certain regions, such as Guinea-Bissau, Egypt, and Nigeria, have reported pooled prevalence rates exceeding 12% [2]. In Korea, despite variations attributed to the study period and testing methods, the prevalence ranges from 1% to 4% [10, 11].

In this study, the annual positivity rate of HAstV remained 2–3% between 2015 and 2019, and then decreased to 0.5% in 2020, followed by an increase to 1.5% in 2021 and 3.6% in 2022. This trend may be due to the fact that strict social distancing and mask wearing were implemented during the COVID-19 pandemic, but social distancing was weakened as the pandemic prolonged [17–19]. Moreover, while HAstV positivity rates did not exhibit distinct seasonal trends in the past, monthly positivity rates surged in 2022, ranging from 6% to 10% between June and August. These findings align with sentinel surveillance data on intestinal infectious diseases from the Korea Disease Control and Prevention Agency [20]. This may be because social distancing during the COVID-19 pandemic has reduced exposure to pathogens and weakened the patient’s immunity, including a decrease in antibody titre.

HAstV-induced AGE is primarily associated with children <5 years old [1]. Consistent with this notion, our study revealed a positivity rate of 7.3% for children aged ≤5 years, more than six times higher than the 1.2% positivity rate in patients with AGE aged >5 years. Additionally, 77.4% of all HAstV-positive samples were found in patients aged ≤5.

The genetic distances among these strains are illustrated in a phylogenetic tree (Figure 2). The strains exhibited clear distinctions from NC001943, the reference strain of HAstV1, and slight variations from the HAstV-1s detected in Korea in 2016 and 2017 (MK472054 and MK430063). Other types of reference strains showed significant genetic distances from the HAstV1 strains.

Other classic HAstV types – such as HAstV-2, HAstV-4, HAstV-5, and HAstV-8, as well as novel HAstV-MLB or HAstV-VA types reported in other studies – were not detected in this study [2]. By contrast, our study identified only one genotype. A 2023 meta-analysis revealed pooled prevalence of 17.4%, 6.7%, 3.5%, 4.5%, and 0.7% for MLB1, MLB2, VA1, VA2, and VA3, respectively [2]. More extensive data collection in the future is essential to discern whether the genotype differences observed between this study and others are specific to the Korean region or indicate post-COVID-19 characteristics. This study is the first in Korea to analyse the non-classic astrovirus types HAstV-MLB and HAstV-VA.

The study had limitations owing to its retrospective nature and the analysis of only a subset of positive samples (Table 1). Nevertheless, the genotyping results indicated a consistent presence of the HAstV-1 genotype with nearly identical nucleotide sequences across all samples, suggesting its prevalence during 2022. Another limitation arose from using Seegene reagents, which detect classical astrovirus types 1–8. Consequently, genotypes such as HAstV-MLB (MLB1–3) and HAstV-VA (VA1–5) may not have been identified. Therefore, additional PCR testing was conducted on 100 negative samples from the same period, using primers capable of identifying these specific genotypes. The negative results inferred the absence of HAstV-MLB and HAstV-VA genotypes during the peak period of HastV-1 from June to August 2022.

Astrovirus, characterized by its relatively low frequency compared to more common rotavirus or norovirus, poses challenges in sample collection and storage, particularly when targeting stool samples. After an epidemic occurs, it is more difficult to find past samples to investigate the cause of the epidemic. However, this study used rare HAstV-positive stool samples collected during the surge of HAstV in the summer of 2022. This study confirmed the astrovirus surge in Korea in the summer of 2022 and revealed that the astrovirus genotypes prevalent in 2022 were all HAstV-1 types with similar nucleotide sequences. Our results provide recent epidemiological data on HAstVs in Korea. The decline and surge in annual astrovirus positivity in recent years may be related to the COVID-19 pandemic.

## Data Availability

The data that support the findings of this study are available upon reasonable request from the corresponding author.
